# A Biomarker Validation Study of Prenatal Chlorpyrifos Exposure within an Inner-City Cohort during Pregnancy

**DOI:** 10.1289/ehp.0800041

**Published:** 2008-12-05

**Authors:** Robin M. Whyatt, Robin Garfinkel, Lori A. Hoepner, Howard Andrews, Darrell Holmes, Megan K. Williams, Andria Reyes, Diurka Diaz, Frederica P. Perera, David E. Camann, Dana B. Barr

**Affiliations:** 1 Columbia Center for Children’s Environmental Health, Mailman School of Public Health, Columbia University, New York, New York, USA;; 2 Southwest Research Institute, San Antonio, Texas, USA;; 3 National Center for Environmental Health, Centers for Disease Control and Prevention, Atlanta, Georgia, USA

**Keywords:** biomarkers, chlorpyrifos, cord blood, indoor air, maternal blood, meconium, pregnancy, urine

## Abstract

**Background:**

We previously documented significant decreases in chlorpyrifos concentrations in maternal personal and indoor air samples among pregnant African-American and Dominican women from New York City after the 2000–2001 restrictions on its residential use.

**Objective:**

We undertook a biomarker validation study within the same cohort to evaluate trends over time in multiple biomarkers of prenatal chlorpyrifos exposure.

**Methods:**

Subjects were enrolled between February 2001 and May 2004 (*n* = 102). We measured 3,5,6-trichloro-2-pyridinol (TCPy) in postpartum meconium (*n* = 83), repeat prenatal maternal spot urine samples (*n* = 253), and postnatal urine from the mothers (*n* = 73) and newborns (*n* = 59). We measured chlorpyrifos in postnatal maternal (*n* = 92) and umbilical cord (*n* = 65) blood.

**Results:**

We did not detect TCPy in infant urine, but all other biomarkers showed a highly significant decrease in detection frequencies (χ^2^ = 7.8–34.0, *p* ≤ 0.005) and mean ranks (*p* ≤ 0.006, Kruskal–Wallis) among subjects enrolled in 2003–2004 compared with those enrolled in 2001–2002. Chlorpyrifos in maternal personal and indoor air declined 2- to 3-fold over the same period (*p* < 0.05). In 2001–2002 samples, TCPy levels in repeat prenatal urine were positively correlated (*r* = 0.23–0.56), but within-subject variability exceeded between-subject variability (intraclass correlation coefficient = 0.43); indoor air levels explained 19% of the variance in prenatal urine TCPy (*p* = 0.001). Meconium TCPy concentrations were positively correlated with chlorpyrifos in maternal and cord blood (*r* = 0.25–0.33, *p* < 0.05) and with TCPy in maternal urine (*r* = 0.31, *p* < 0.01).

**Conclusions:**

Results suggest the biomarkers are reliable dosimeters to differentiate between groups with prenatal chlorpyrifos exposures varying by a factor of 2 or more and vividly illustrate the efficacy of residential restriction on chlorpyrifos to reduce the internal dose during pregnancy.

Residential pesticide use is widespread in the United States. Eighty-five percent of American households store at least one pesticide in their home, and approximately 10% of the mass of conventional pesticides used annually in the United States are applied in and around the home (Adgate et al. 2000; Kiely et al. 2004). Until its use was restricted, chlorpyrifos was one of the most widely used insecticides for residential pest control. Use was particularly heavy in New York City. During 1997, the amount of the insecticide applied by licensed applicators in New York City exceeded the amount applied in any other county in New York State, including agricultural communities (Thier 1998). In June 2000, the U.S. Environmental Protection Agency (EPA) entered into an agreement with the registrant to begin phasing out residential chlorpyrifos uses and to terminate all retail sales for indoor use by December 2001 (U.S. EPA 2000). Chlorpyrifos is still widely used in agriculture, with approximately 10 million pounds applied annually in the United States; use on corn constitutes the largest market share (U.S. EPA 2002).

We have previously reported on chlorpyrifos concentrations in personal and indoor air samples among a cohort of 102 women residing in inner-city communities within New York City (Whyatt et al. 2007). Chlorpyrifos was detected in 99% of 48-hr maternal personal air samples during the 32nd week of pregnancy and in 99.7% of 2-week integrated indoor air samples collected sequentially from the 32nd week of pregnancy until delivery. There was little within-home variability in indoor air levels; between-home variability accounted for 92% of the variance in chlorpyrifos air concentrations. However, indoor and personal air samples declined significantly after the residential phaseout of chlorpyrifos use: from an average of 12.4 ng/m^3^ for indoor air and 9.3 ng/m^3^ for personal air among subjects monitored in 2001 to an average of 2.4 and 2.2 ng/m^3^, respectively, among subjects monitored in 2004. Here we report of a biomarker validation study within the same cohort.

Biological markers of chlorpyrifos have been used extensively in prior research to assess the extent and pathways of exposure in both children and adults (Clayton et al. 2003; Curl et al. 2002; Lu et al. 2006; Morgan et al. 2005; Pang et al. 2002) and to evaluate the effects of exposure on fetal and postnatal development (Berkowitz et al. 2004; Engel et al. 2007; Eskenazi et al. 2004, 2007; Rauh et al. 2006; Whyatt et al. 2004). In general, biomonitoring studies of contemporary pesticide exposures employ urine as the matrix because of ease of collection (Barr and Angerer 2006). The most common urinary biomarker of chlorpyrifos is 3,5,6-trichloro-2-pyridinol (TCPy). TCPy is also a metabolite of chlorpyrifos-methyl and triclopyr (Morgan et al. 2005). Additionally, TCPy can be derived from exposure to environmental degradation products of chlorpyrifos (Barr and Angerer 2006). Prior biomonitoring studies have also measured chlorpyrifos or its metabolite in blood and meconium (Whyatt and Barr 2001; Whyatt et al. 2003). However, no prior study has measured all of these biomarkers simultaneously within one cohort over a period when exposures were declining rapidly. The present biomarker validation study is nested within the longitudinal birth cohort study of 725 African-American and Dominican mothers and newborns from New York City being conducted by the Columbia Center for Children’s Environmental Health (CCCEH). It includes measurements of chlorpyrifos or its metabolite in urine, blood, and meconium collected during pregnancy and after delivery from 102 mothers and newborns enrolled into the CCCEH cohort between 2001 and 2004. The primary purpose of this study was to assess whether chlorpyrifos biomarkers declined after the phaseout of residential uses. We also undertook this study to evaluate the interrelationship between the biomarkers and to evaluate the within- and between-subject variability in TCPy levels in repeat spot urine samples collected from the mothers during pregnancy. Chlorpyrifos has a short biological half-life in blood, adipose tissue, and urine of an estimated 24, 62, and 15–30 hr, respectively (Barr and Angerer 2006). Previous research has documented significant intraindividual variability in urinary metabolite levels in children and adults (MacIntosh et al. 1999; Meeker et al. 2005), but no prior studies have assessed variability in TCPy levels in repeat spot urine samples collected from women during pregnancy.

## Materials and Methods

The study includes 102 African-American and Dominican women selected from the CCCEH cohort. Enrollment criteria for the CCCEH cohort have been described elsewhere (Perera et al. 2003; Whyatt et al. 2002, 2003). Women were enrolled through the prenatal clinics at New York Presbyterian and Harlem Hospital Centers in New York City between 2001 and 2004. We restricted the study to women 18–35 years old who self-identified as African American or Dominican and had resided in northern Manhattan (Central Harlem or Washington Heights/Inwood) or the South Bronx for ≥ 1 year before pregnancy. Women were excluded if they smoked cigarettes or used other tobacco products during pregnancy, used illicit drugs, had diabetes, hypertension, or known HIV, or had their first prenatal visit after the 20th week of pregnancy. All women were registered to deliver at New York Presbyterian or Harlem Hospital Center. In addition to the enrollment criteria for the full CCCEH cohort, we restricted enrollment into the present study to women who were not employed outside the home at the time of enrollment, to eliminate confounding from pesticide exposures in the workplace. Workplace exposures would be difficult for the woman to reliably quantify. We explained study procedures, including the personal and indoor air monitoring and collection of biological samples, to each subject at enrollment. We scheduled the personal and indoor air monitoring to commence around the 32nd week of pregnancy. The study was approved by the Institutional Review Board at Columbia University, and all study subjects gave written informed consent before participating.

As described previously (Whyatt et al. 2007), the women in the present study were comparable with women in the remaining CCCEH cohort in terms of maternal age, ethnicity, marital status, and annual household income, and a similar proportion reported using pest control methods during pregnancy. However, a greater proportion of women in the present study compared with those in the remaining CCCEH cohort reported less than a high school education (46% vs. 34%; χ^2^ = 5.8, *p* = 0.02), and a greater proportion of the women in the remaining CCCEH cohort compared with those in the present study reported employment at some point during pregnancy (55.7% vs. 41%; χ^2^ = 7.4, *p* < 0.01). Most of the women (96%) in the present study lived in residential apartment buildings; the remaining lived in multifamily housing (*n* = 3) or in combined residential/commercial buildings (*n* = 1).

### Questionnaire data

We administered a 45-min questionnaire to the women at the first home visit during the 32nd week of pregnancy. It included basic demographic information, neighborhood and home characteristics, history of active and passive smoking, and history of exposures, including pesticide use during pregnancy. In addition, at each biweekly home visit, we administered a short 10–15 min questionnaire to gather exposure information specific to pesticide exposures during the preceding 2 weeks. Questions included whether pests (specifically cockroaches, mice, rats, or other pests) were seen in the home, frequency of sightings over the 2-week period, and whether any pest control measures were used over the prior 2 weeks either by an exterminator or by others in the home (the participant, building superintendent, or other household member). If pest control measures were used, we gathered information on which methods were used, who used them, what pest they were used for, and how frequently they were used. Because 47% of the cohort spoke Spanish, the interviews were conducted in both Spanish and English by a fully bilingual research staff. We gathered a total of 333 2-week questionnaires from the study participants; 74% of women completed three or more questionnaires.

### Indoor and personal air sampling

The indoor air monitoring was conducted continuously beginning during the 32nd week of pregnancy until delivery. We collected 2-week integrated indoor air samples every 2 weeks throughout the monitoring period. We monitored the air using a pump with a 0.5-L/min flow rate attached to a polyurethane foam (PUF) sampler with a 2.5-μm inlet cut fitted with a 25-mm quartz fiber filter and a foam cartridge backup to capture semivolatile vapors and aerosols, as previously described (Whyatt et al. 2007). The pump was attached to a battery and operated at 0.5 L/min continuously throughout the 2 weeks. Every other week, the PUF sampler was removed and a new sampler and battery attached. The indoor air monitoring continued until the woman went into labor, for an average of 7.0 ± 2.3 (range, 1.9–12.4) weeks per home, with an average of 3.5 ± 1.1 (range, 1–6) 2-week integrated indoor air samples collected per home. The monitoring continued for ≥ 6 weeks in 80% of the homes. In four cases, we collected an additional 2-week integrated indoor air sample after delivery because predelivery samples had been lost through laboratory error. We collected the personal air sample from the mother over 48 hr during the 32nd week of pregnancy. We asked women in the cohort to wear a small backpack containing a personal ambient air monitor during the daytime hours for 2 consecutive days and to place the monitor near the bed at night. The personal air sampling pump operated continuously at 4 L/min over this period, collecting particles of ≤ 2.5 μm in diameter on a quartz microfiber filter and collecting semivolatile vapors and aerosols on a PUF cartridge backup. We collected a 48-hr personal air sample from 96 of 102 (94%) of the women in the study. We initiated the indoor sampling at the conclusion of the 48-hr personal air sampling.

We processed and analyzed indoor and personal air samples as described previously (Whyatt et al. 2007). Briefly, immediately after collection, the indoor and personal air monitoring samples were brought to the molecular epidemiologic laboratory at the Mailman School of Public Health, inventoried, and frozen. Once per month, air samples were shipped on dry ice to Southwest Research Institute and stored at − 12°C. Within 10 days of arrival, the PUF plug and filter were placed in a Soxhlet extractor (Corning, Corning, NY), spiked with terphenyl-d_14_ as a recovery, and extracted with 6% diethyl ether in hexanes for 16 hr. We then concentrated the extract to 1 mL and froze it at − 12°C before analysis. We analyzed chlorpyrifos by gas chromatography/mass spectrometry (GC/MS) as described previously (Whyatt et al. 2007).

### Urine sample collection

We collected repeat prenatal maternal spot urine samples from women at the end of each 2-week indoor air sampling, beginning at the 34th week of pregnancy and continuing until delivery. We did not collect urine from women who went into labor before the end of the first 2 weeks of indoor air sampling. We collected a total of 253 repeat prenatal spot urine samples from 97 of 102 women (average of 2.6 samples per woman): 53 of 97 (53%) of the women provided three or more urine samples, 29 of 97 (30%) two samples, and 15 of 97 (15%) provided one sample. We also collected a urine sample the day after delivery from 73 women and 59 newborns. The prenatal urine collection took place in the woman’s home. The postnatal urine collection took place in the hospital. All maternal urine samples were collected using sterile cups supplied by the Centers for Disease Control and Prevention (CDC). Newborn urine samples were collected using sterile newborn urine collection bags. Immediately after collection, the urine samples were transported to the laboratory, inventoried, and frozen at − 80°C before shipment to CDC for analyses. Before freezing, a 2-mL sample of urine was drawn off and frozen separately for creatinine analysis.

### Blood sample collection

A sample of umbilical cord blood was collected as soon after delivery as possible by drawing the blood into a heparinized syringe to avoid clotting. A sample of maternal blood was obtained within 2 days postpartum into heparinized Vacutainer tubes by the hospital staff. An umbilical cord blood sample was collected from 65 newborns, and a maternal blood sample was collected from 92 women; paired blood samples were available for 64 of the mothers and newborns. Within 12 hr of collection, the cord and maternal blood samples were transferred to a centrifuge tube and spun for 15 min at 1,500 rpm. The plasma was collected and stored at − 80°C before shipment on dry ice to CDC.

### Collection of postpartum meconium

Meconium samples were collected from 83 newborns during the postpartum hospital stay. In 53 (64%) of the births, the meconium samples were collected within 1 day of delivery; in 19 (23%) of the births, within 2 days after delivery; and for 10 (12%) of the births, within 3 days after delivery; for one birth, the date of meconium collection was not recorded. The meconium was collected by transferring the sample from the diaper into a urine cup supplied by CDC. The sample was transported to the laboratory and stored at − 80°C before shipment to CDC for pesticide analyses.

### Analyses of pesticides and creatinine in biological samples

All biological samples were shipped on dry ice to CDC twice per year. Analysis of the pesticides or their metabolites in biological samples was conducted by the CDC using previously established methods (Barr et al. 2002; Olsson et al. 2004). Briefly, we spiked plasma samples (4 g) with stable isotopically labeled chlorpyrifos and mixed them well. We denatured the samples with saturated ammonium sulfate and extracted the supernatant using mixed-polarity solid-phase extraction cartridges. We concentrated the eluate (in dichloromethane) and analyzed it using gas chromatography/high-resolution mass spectrometry at 10,000 resolving power (Barr et al. 2002) with both quantification and confirmation ions monitored. The urine samples (2 mL) were spiked with stable isotopically labeled TCPy, mixed well, and then subjected to an enzyme hydrolysis to liberate glucuronide- and sulfate-bound TCPy. We extracted the hydrolysates using mixed-polarity solid-phase extraction cartridges. The eluates (methanol) were concentrated and analyzed using HPLC/tandem MS (Olsson et al. 2004) with both quantification and confirmation ions monitored. We homogenized thawed meconium samples before analysis, because the pesticides may not be evenly distributed through the meconium. We extracted approximately 0.5–1 g of meconium with 5 mL methanol. We concentrated the methanol and then analyzed it using HPLC/tandem MS according to the analytical parameters of the modified method of Olsson et al. (2004).

We quantified chlorpyrifos and TCPy, irrespective of matrix, using isotope dilution calibration. The limit of detection (LOD) of chlorpyrifos in blood samples was 0.5–1 pg/g plasma. The LOD of TCPy in urine samples was 0.26 ng/mL urine. The LOD for TCPy in meconium was 0.2 ng based on a sample weighing 0.5 g. Meconium samples analyzed in the present study weighed generally between 0.5 and 0.7 g. Details on quality control and reproducibility for analyses of chlorpyrifos in blood and TCPy in urine have been published previously (Barr et al. 2002; Olsson et al. 2004). The relative standard deviation of TCPy in the meconium quality control pools was 16%.

We performed the urine creatinine measurements according to the Roche Creatinine Plus Assay using a Roche Hitachi Automatic Analyzer model 912 (Roche Diagnostics, Indianapolis, IN). The Creatinine Plus Assay relies on the chemistry of creatinase and sarcosine oxidase. Through a series of chain reactions in which creatinine is converted to creatine, which is in turn converted to sarcosine and urea, sarcosine is oxidized and hydrogen peroxide is formed. The peroxidase-catalyzed oxidation of a leuko dye by hydrogen peroxide produces a red color whose intensity is measured through absorbance readings and is directly proportional to the creatinine concentration in the sample.

Approximately 10% of all samples analyzed for chlorpyrifos, TCPy, or creatinine were positive or negative control samples. We used two concentrations of positive controls samples: one spiked at the mid-calibration range and one at the low calibration range. All positive control samples were evaluated according to the Westgard multirules of quality control (Westgard 2002) by an independent quality assurance officer as well as laboratory personnel.

### Statistical analyses

Descriptive analyses preceded formal hypothesis testing. We calculated the percentage of biological samples with chlorpyrifos levels above the LOD. Before statistical analyses, pesticide values were log-transformed as needed to normalize their distributions. We assigned values below the LOD a value of half the LOD. We used nonparametric statistics if distributions could not be normalized after transformations. With nonparametric statistics, data are ranked and the statistical tests compare correlations or differences in mean ranks between groups rather than differences in concentrations. Because no assumptions are made about the distribution of the data, nonparametric statistics are appropriate when a large proportion of the data are below the LOD (as was frequently the case in our study). When ranked values have the same numeric number (as is the case with values below the LOD), the rank is 0.5 times the number of ties. We used Spearman’s rank correlation coefficients to examine correlations between levels of chlorpyrifos or TCPy in the biological samples and to examine correlations between levels of chlorpyrifos or TCPy in the biological samples and chlorpyrifos levels in the indoor air samples. We used multiple linear regression to determine the amount of variance explained in prenatal maternal urine TCPy levels by the indoor air chlorpyrifos levels among subjects enrolled in 2001–2002. We detected TCPy too infrequently to conduct analyses among subjects enrolled after 2002. We used analysis of variance or Kruskal–Wallis (depending on whether parametric or nonparametric analyses were appropriate) to test whether pesticide levels varied by year of monitoring (2001–2004) or among the following groups: women not using any pest control methods; women using nonspray methods only (sticky traps, bait traps, boric acid, and gels); and women using can sprays, pest bombs, or sprays by an exterminator, with or without nonspray methods. If levels differed significantly among the groups, we used the least significant difference test or Mann–Whitney *U*-test to determine which groups varied significantly. We used group *t*-test or Mann–Whitney *U*-test to test whether pesticide levels differed significantly by ethnicity. We used chi-square analyses to test whether the frequencies of detection of chlorpyrifos to TCPy in the biological samples varied by year of the monitoring. We also used the chi-square test to compare detection frequencies of chlorpyrifos in maternal and cord blood samples. We used a mixed effects model to evaluate the within- and between-subject variability in TCPy levels in the repeat maternal urine samples collected during pregnancy, and to calculate the intraclass correlation coefficient (ICC). The ICC is a measure of reliability of repeated measures over time, defined as the ratio of between-subject variance to total variance. For these analyses we included women who had three repeat urine samples; we also restricted analyses to subjects enrolled in 2001–2002 because TCPy was detected in only one-third of samples among subjects enrolled after 2002 and the distributions could not be normalized.

Results were considered significant at *p* < 0.05. We performed analyses using SPSS (version 16.0; SPSS, Chicago, IL) except for the mixed effects models, for which we used PROC MIXED in SAS version 9.3 (SAS Institute Inc., Cary, NC).

## Results

Table 1 provides demographics for the cohort and gives the number of women who reported sighting of pests in the home or use of pest control measures between the 32nd week of pregnancy and delivery. Ninety (91%) of the women reported that they sighted pests; cockroaches were the pests sighted most frequently. Fifty-nine (60%) of the women reported using some form of pest control during the study period: 31 women (32%) reported using baits, gels, and traps only, and 28 (29%) reported using one or more of the spray methods (can sprays, sprays by exterminator, and pest bombs) with or without the other methods. There was no difference in self-reported pesticide use between African Americans and Dominicans. The women reported spending an average of 21.3 ± 1.75 hr (range, 14–24 hr) per day at home between the 32nd week of pregnancy and delivery.

**Figure 1:**
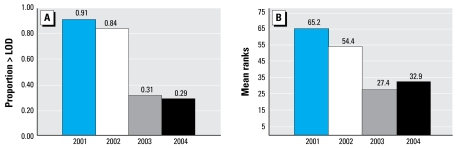
Percentage of subjects with TCPy concentrations in one or more of repeat maternal prenatal urine samples > LOD (*A*) and mean TCPy ranks adjusted for creatinine (*B*) by year of sample collection. Sample size: 2001, *n* = 32; 2002, *n* = 25; 2003, *n* = 26; 2004, *n* = 14. (*A*) Chi-square, 2001–2002 versus 2003–2004 = 34.0 (*p* < 0.001). (*B*) Mean ranks: 2001–2002 versus 2003–2004, 60.5 versus 29.3, *p* < 0.001, Kruskal–Wallis; 2001 versus 2002, *p* = 0.04; 2001 versus 2003, *p* < 0.001; 2001 versus 2004, *p* = 0.005; 2002 versus 2003, *p* < 0.001; 2002 versus 2004, *p* = 0.01, Mann–Whitney *U*-test.

Table 2 provides levels of TCPy in repeat spot urine samples collected from the mother between the 34th week of pregnancy and delivery. We detected TCPy in one or more of the repeat prenatal urine samples collected from 62 of 97 (64%) of the mothers. Mean TCPy concentration in prenatal urine was 1.4 ± 1.8 μg/g creatinine. We also detected TCPy in 42% of the postnatal urine sample (range, < LOD to 8.7 μg/g creatinine). We did not detect TCPy in any of the urine samples collected from the newborns after delivery. Figure 1 shows detection frequencies and mean ranks of TCPy in the repeat prenatal urine samples by the year women enrolled into the study. Detection frequencies for TCPy dropped significantly between subjects enrolled in 2001–2002 and those enrolled in 2003–2004 for both prenatal (χ^2^ = 34.0, *p* < 0.001; Figure 1) and postnatal (χ^2^ = 20.3, *p* < 0.001; data not shown) maternal urine samples. Mean ranks, adjusted for creatinine, also decreased significantly from 65.2 in 2001 to 32.9 in 2004 for prenatal urine samples (*p* < 0.001; Figure 1) and from 42.1 to 23.9 for postnatal urine (*p* < 0.001; data not shown). The difference in mean ranks for subjects enrolled in 2001–2002 compared with those enrolled in 2003–2004 was highly significant for both average maternal TCPy in urine during pregnancy (60.5 vs. 29.3; *p* < 0.001) and after delivery (42.1 vs. 23.9; *p* < 0.001). In addition, we found a significant difference in mean ranks between subjects enrolled in 2001 and 2002 but no difference in mean ranks between subjects enrolled in 2003 and 2004.

By comparison, among subjects enrolled in 2001–2002 compared with 2003–2004, chlorpyrifos levels in the 2-week integrated indoor air samples also decreased from 10.0 to 3.2 ng/m^3^ (*p* = 0.002) and in maternal 48-hr personal air samples from 8.1 to 3.7 ng/m^3^ (*p* = 0.04; data not shown). TCPy levels in the repeat maternal urine samples during pregnancy were significantly higher among African-American than among Dominican women (mean ranks, 56.5 and 41.9, respectively; Mann–Whitney *U*-test, *p* = 0.02). We found no difference in TCPy levels in maternal urine samples collected after delivery between African Americans and Dominicans, and no association between TCPy in maternal urine samples during pregnancy or after delivery and maternal self-reported pesticide use (data not shown).

Table 3 shows the correlation between the TCPy levels, adjusted for creatinine, in the repeat maternal urine samples collected during pregnancy and after delivery, both for all subjects and for subjects enrolled in 2001–2002. The correlations were generally positive and often significant. Among women enrolled in 2001–2002, the ICC for TCPy in the repeat prenatal urine samples adjusted for creatinine was 0.43. Results were similar after adjusting for ethnicity. Among women enrolled after 2002, we detected TCPy too infrequently in urine samples to determine the ICC; however, we found no significant correlation between TCPy levels in the repeat urine samples (*r*-values ranged from − 0.2 to 0.2, *p*-values > 0.4; data not shown).

Table 4 shows the correlation between chlorpyrifos in the repeat 2-week integrated indoor air samples and TCPy levels in the repeat maternal spot urine samples collected over the same period. The correlations were positive and statistically significant in most cases. This was true both among all subjects and among subjects enrolled in 2001–2002. Among subjects enrolled in 2001–2002, average indoor air chlorpyrifos levels over the final 2 months of pregnancy explained 19% of the variance in average TCPy levels in maternal repeat prenatal spot urine (*p* = 0.001). Results were similar after controlling for ethnicity. We found no association between chlorpyrifos in repeat indoor air samples and TCPy in the repeat maternal urine samples among subjects enrolled after 2002.

**Figure 2:**
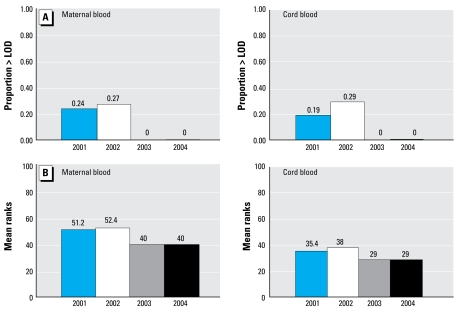
Percentage of maternal and umbilical cord blood levels with chlorpyrifos concentrations > LOD (*A*) and mean chlorpyrifos ranks (*B*) by year of sample collection. Maternal blood sample size: 2001, *n* = 29; 2002, *n* = 22; 2003, *n* = 26; 2004, *n* = 15. Cord blood sample size: 2001, *n* = 21; 2002, *n* = 14; 2003, *n* = 20; 2004, *n* = 10. (*A*) In maternal blood, 2001–2002 versus 2003–2004, χ^2^ = 12.2 (*p* < 0.001); in blood, 2001–2002 versus 2003–2004, χ^2^ = 7.8 (*p* = 0.005). (*B*) Mean ranks, maternal blood: 2001–2002 versus 2003–2004, 51.7 versus 40.0, *p* = 0.001, Kruskal–Wallis; 2001 versus 2003, *p* = 0.008; 2001 versus 2004, *p* = 0.04; 2002 versus 2003, *p* = 0.005, Mann–Whitney *U*-test. Mean ranks, cord blood, 2001–2002 versus 2003–2004, 36.4 versus 29.0, *p* = 0.006, Kruskal–Wallis; 2001 versus 2003, *p* = 0.04, Mann–Whitney *U*-test.

**Figure 3:**
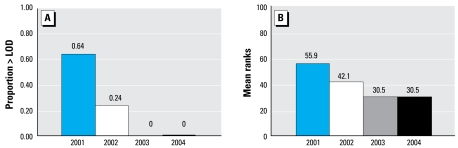
Percentage of meconium samples with TCPy concentrations > LOD (*A*) and mean TCPy ranks (*B*) by year of sample collection. Sample size: 2001, *n* = 28; 2002, *n* = 21; 2003, *n* = 21; 2004, *n* = 13. (*A*) For samples > LOD, 2001–2002 versus 2003–2004, χ^2^ = 22.1, *p* < 0.001. (*B*) Mean ranks, 2001–2002 versus 2003–2004, 50.0 versus 30.5, *p* < 0.001, Kruskal–Wallis; 2001 versus 2003, *p* < 0.001; 2001 versus 2004, *p* = 0.001; 2002 versus 2003, *p* = 0.02, Mann–Whitney *U*-test.

Table 5 provides detection frequencies and levels of chlorpyrifos in maternal and umbilical cord blood and TCPy in meconium collected from subjects after delivery. Figures 2 and 3 show detection frequencies and mean ranks by year of enrollment. We detected TCPy in meconium more frequently and at higher levels than chlorpyrifos in the maternal or cord blood. Consistent with the trends we found with chlorpyrifos in the indoor and personal air samples and with TCPy in maternal urine samples, we found a highly significant difference in detection frequencies and mean ranks by year of enrollment (Figures 2 and 3). The difference was seen only between subjects enrolled in 2003–2004 and those enrolled in 2001–2002. We found no significant difference in biomarker levels between subjects enrolled in 2001 compared with 2002 or in 2003 compared with 2004. However, between 2001–2002 and 2003–2004, detection frequencies decreased significantly for TCPy in meconium (χ^2^ = 22.1, *p* < 0.001) and chlorpyrifos in maternal blood (χ^2^ = 12.2, *p* < 0.001) and umbilical cord blood (χ^2^ = 7.8, *p* = 0.005). By 2003, TCPy was no longer detected in any meconium and chlorpyrifos was not detected in any blood samples. Between 2001–2002 and 2003–2004, mean ranks also decreased significantly from 50.0 to below the LOD for TCPy in meconium (*p* < 0.001) and from 51.7 and 36.4 to below LOD chlorpyrifos in maternal (*p* = 0.001) and umbilical cord blood (*p* = 0.006), respectively (Kruskal–Wallis).

Chlorpyrifos in blood and TCPy in meconium were not associated with personal and indoor air chlorpyrifos levels, nor did they vary by maternal self-reported pesticide use or by ethnicity (data not shown). Further, we found no association between chlorpyrifos levels in maternal and cord blood and TCPy levels in maternal urine samples during pregnancy or after delivery. However, TCPy levels in meconium were weakly but significantly correlated with chlorpyrifos levels in maternal (*r* = 0.25. *p* = 0.03) and umbilical cord (*r* = 0.33, *p* = 0.01) blood (Table 6) and with average TCPy levels in maternal urine samples during pregnancy (*r* = 0.31, *p* = 0.006) and after delivery (*r* = 0.32, *p* = 0.01; Table 6). Maternal and umbilical cord blood levels were highly correlated (*r* = 0.9, *p* < 0.001, *n* = 64). Of the 10 paired samples where the mother’s levels were > LOD, the cord blood was > LOD in 8 of 10; of the 54 samples when the mother’s level was < LOD, the cord was also always < LOD (χ^2^ = 49.4, *p* < 0.001). When analyses were restricted to the 10 pairs where the mother’s levels were > LOD, the correlation between maternal and cord blood levels remained statistically significant (*r* = 0.71, *p* = 0.02).

## Discussion

The present study provided a unique opportunity to validate a battery of biomarkers of prenatal chlorpyrifos exposure, because we began the study after the phaseout of residential use of chlorpyrifos and during a time when exposure levels among the CCCEH cohort were decreasing rapidly (Whyatt et al. 2007). Our prior research has shown that residential pesticide use is widespread among cohort women during pregnancy (Whyatt et al. 2005). Further, until use was restricted in 2001, chlorpyrifos was one of the most widely used insecticides for pest control in New York City (Thier 1998). However, after the restriction we documented a highly significant decrease in chlorpyrifos levels in personal and indoor air levels from the women during pregnancy (Whyatt et al. 2007). In preliminary analyses, we also saw a significant decrease in chlorpyrifos levels in maternal and umbilical cord blood among subjects enrolled between 1999 and 2002 (Whyatt et al. 2005). We undertook the present study in a subset of 102 mothers and newborns from the CCCEH cohort enrolled over a longer period. In addition to measuring chlorpyrifos in maternal and umbilical cord blood samples, we also measured levels of TCPy in repeat spot urine samples collected biweekly from the mother over the final 6–8 weeks of pregnancy, and in urine samples collected from both mother and newborn after delivery and TCPy in postpartum meconium. We did not detect TCPy in newborn urine. However, we saw a highly significant decrease in the detection frequency and mean ranks of all of the other biomarkers among subjects enrolled during years 2001–2004.

Both maternal and umbilical cord blood chlorpyrifos levels were below the LOD in all samples collected after 2002. We have previously reported that chlorpyrifos levels in umbilical cord blood among infants born in the cohort in 1999 averaged 6.9 pg/g and averaged 3.5 pg/g among those born in 2000 (Whyatt et al. 2005). Here we report that we detected chlorpyrifos in only 19–29% of maternal and cord blood samples in 2001–2002 but not in any blood samples collected in 2003–2004. Collectively, these results suggest chlorpyrifos from residential use was the main contributor to the chlorpyrifos levels we have detected in both the maternal and umbilical cord blood among study subjects. Although it is likely that the women continued to receive some chlorpyrifos exposures after 2002, including exposure from the diet (Lu et al. 2006), any remaining levels were below the LOD of the blood measurement. Consistent with our previous analyses (Whyatt et al. 2004), maternal and cord chlorpyrifos levels were highly correlated. It has been speculated that this correlation might have occurred because the amount of chlorpyrifos in maternal blood is in equilibrium with the amount in adipose tissue (Needham 2005) and chlorpyrifos is readily transferred from maternal to cord blood across the placental (Whyatt et al. 2003). However, in our case the correlation was being driven principally by the concordance of maternal and cord blood samples below the LOD.

We saw a similar and significant decrease in TCPy levels in meconium among subjects enrolled between years 2001 and 2004. As with the maternal and umbilical cord blood samples, levels of TCPy in meconium were above the LOD among infants born in 2001–2002 but not in 2003–2004. However, before 2003, TCPy was detected more frequently and at much higher levels in meconium than chlorpyrifos in either maternal or umbilical cord blood samples. For example, among subjects enrolled in 2001, 64% of meconium samples had detectable TCPy levels with a mean of 0.43 ± 0.21 ng/g, whereas only 24% and 19% of maternal and umbilical cord blood samples had detectable levels of chlorpyrifos, with a range up to 3.0 pg/g. Further, TCPy levels in meconium were significantly correlated with chlorpyrifos levels in both maternal and umbilical cord blood and with TCPy levels in maternal urine samples, whereas we saw no association between chlorpyrifos in maternal and umbilical blood samples and TCPy in maternal urine samples. These results suggest that TCPy in meconium may provide a valid biomarker of prenatal exposure.

There has been considerable interest in the measurement of nonpersistent pesticides in meconium as a biomarker for exposures during pregnancy (Bearer 2003; Ostrea et al. 2008; Wessels et al. 2003; Whyatt and Barr 2001). In human fetuses, meconium begins to accumulate in the bowels at approximately 16 weeks gestation and is generally not excreted until after delivery (Moriya et al. 1994). It has been hypothesized that xenobiotics enter the meconium as a consequence of bile excretion into the intestines and/or of swallowing by the fetus of amniotic fluid (Ostrea 1999). Other mechanisms may be operating, as well (Mahone et al. 1994). Several lines of evidence suggest that the half-life of xenobiotics in meconium may be protracted and measured levels can reflect exposures from the second trimester of pregnancy through delivery (Bearer et al. 1999, 2003). Most of the epidemiologic research to date has involved analysis of illicit drugs, alcohol, and tobacco products in meconium. Several studies suggest that meconium measurements of these compounds are a more sensitive biomarker than maternal urine or cord bloods measurements (Bearer et al. 2003; Browne et al. 1994; Dempsey et al. 1999; Konkol et al. 1994; Lewis et al. 1995; Maynard et al. 1991; Moore et al. 1998; Nair et al. 1994; Ostrea 1999; Ryan et al. 1994; Wingert et al. 1993) Dose-response relationships have been detected between illicit drug use, cigarette smoking, and alcohol consumption during pregnancy and metabolite levels in meconium. Several studies have used concentrations of xenobiotics in meconium as a dosimeter to evaluate effects of prenatal exposure on pregnancy outcome (Bearer et al. 2003; Delaney-Black et al. 1996; Mirochnick et al. 1995; Nuesslein et al. 1999; Ostrea 1999; Peterson et al. 2008; Tronick et al. 1996). For example, cotinine in meconium was significantly associated with infections of the lower respiratory tract during the first 6 months, whereas no association was seen with self-reported parental tobacco consumption (Nuesslein et al. 1999). Fatty acid ethyl ester levels in meconium have been associated with maternal alcohol consumption during pregnancy (Bearer et al. 2003) and with adverse infant neurodevelopment post-natally (Peterson et al. 2008). Although there have been relatively few studies of pesticides or their metabolites in meconium (Wessels et al. 2003), both organochlorines (Hong et al. 2002; Zhao et al. 2007) and nonpersistent pesticides have been detected (Bielawski et al. 2005; Ostrea et al. 2008; Whyatt and Barr 2001). As was done here, most prior studies have collected meconium directly from the newborn diaper, which raises concerns about potential contamination of the meconium by xenobiotics contained in infant urine. However, that does not appear to be the case in the present study because we did not detect TCPy in any of the paired newborn urine samples. To our knowledge, the present study is the first to show a correlation between levels of an organophosphate insecticide in meconium and in other biological matrices collected during pregnancy. Additionally, this is the first study to reflect a decrease in insecticide levels in meconium after restrictions on residential use. These results suggest that meconium has promise as a biomarker for assessing effects of prenatal chlorpyrifos exposures on the developing fetus. This is encouraging, because meconium is a more readily accessible biological matrix than umbilical cord blood, given that the latter has to be collected within minutes of delivery, whereas meconium is excreted over several days postpartum. Further, the fact that we saw a higher correlation between chlorpyrifos in maternal and cord blood than between chlorpyrifos in maternal and cord blood and TCPy in meconium does not necessarily detract from the reliability of the meconium biomarker, given that the time frames of exposure represented by the biomarkers are different (days in the case of the blood measures and potentially months in the case of meconium). However, additional validation research is clearly needed. It particular, no prior studies have evaluated the relationship between TCPy in meconium and postnatal outcomes. However, validation of biomarkers includes both the backward process of associating a biomarker with exposure and the forward process of linking a biomarker with effect (Bearer 1998). In contrast, our prior research has shown significant associations between chlorpyrifos concentrations in umbilical cord blood, and newborn birth weight and length (Whyatt et al. 2004) and child mental and motor development at age 36 months (Rauh et al. 2006).

Because of ease of collection, prior biomonitoring studies of nonpersistent pesticides have generally collected urine samples rather than blood or meconium samples (Bradman and Whyatt 2005; Wessels et al. 2003). Most use spot urines rather than 24-hr or first morning void samples (Bradman and Whyatt 2005). However, a recent study measured TCPy and other nonpersistent pesticides in four urine samples collected from 13 children over 24 hr in two different seasons and found levels measured in first morning void samples were the best predictor of weighted average daily metabolite concentrations (Kissel et al. 2005). Consistent with our findings, prior studies have documented significant intraindividual variability in TCPy levels in repeat spot urine samples collected from the same individual. A study by Meeker et al. (2005), for example, measured TCPy in nine repeat urine samples collected from 10 men over 3 months; ICCs ranged from 0.15 to 0.21 before and after adjustment for creatinine or specific gravity, indicating within-subject variability accounted for 79–85% of total variability. Another study that measured TCPy in up to six repeat urine samples collected over 1 year from 80 adults in Maryland showed that the average range of concentrations from the same individual was approximately 50% greater than the respective mean population levels (MacIntosh et al. 1999). However, interindividual variability was found to be significantly greater than intraindividual variability in a study measuring TCPy levels in one to three first morning void urine samples collected from 102 children in Minnesota (Adgate et al. 2001). In the present study, the ICC for TCPy in repeat urine samples from women enrolled in 2001–2002 was 0.43, adjusting for creatinine; thus, 57% of total variability was explained by within-subject variability. We detected TCPy too infrequently in urine samples among women enrolled after 2002 to calculate the ICC.

Despite the significant within-subject variability in urinary TCPy levels seen in the present study, our ICCs were considerably higher than those seen in the Meeker et al. (2005) study described above. Before 2003, indoor air chlorpyrifos levels were a significant predictor of TCPy in repeat maternal urine samples accounting for 19% of the variance in urinary TCPy. Because chlorpyrifos levels remained remarkably stable within most homes over this period, and between-home variability accounted for 92% of the variance in indoor air chlorpyrifos levels (Whyatt et al. 2007), the constant source of exposure may have reduced variability seen in urinary TCPy levels in this cohort relative to other cohorts. However, the low ICCs seen here coupled with those seen in prior studies do raise considerable concern over the reliability of TCPy in a single spot urine sample as a biomarker of exposure. It is also not known the extent to which TCPy in urine reflects exposure to chlorpyrifos or to TCPy itself in the environment (Bradman and Whyatt 2005; Morgan et al. 2005).

Nonetheless, our results suggest that all of the chlorpyrifos biomarkers assessed in the present study do provide reliable internal dosimeters to differentiate between groups having prenatal chlorpyrifos exposures varying by a factor of 2- to 3-fold. Specifically, we saw a highly significant difference in levels of all of the detected biomarkers (chlorpyrifos in blood samples and TCPy in maternal urine and meconium samples) among study subjects enrolled in 2001–2002 compared with those enrolled in 2003–2004. Between these two periods, chlorpyrifos in personal air samples declined approximately 2-fold, and chlorpyrifos in 2-week integrated indoor air samples declined approximately 3-fold. The biomarkers may also provide valid internal dosimeters to differentiate between groups having smaller exposure differentials, but our data do not permit us to assess this possibility. Our sample size in any year was small, exposures were dropping rapidly and the principal difference in biomarker levels seen in the present study was between subjects enrolled in 2001–2002 and 2003–2004.

Limitations in this conclusion also need to be recognized. We have used the differential in chlorpyrifos in personal and indoor air samples as an estimate of the change in chlorpyrifos exposures among women in the cohort. However, we did not conduct an aggregate exposure assessment and so cannot directly test the accuracy of this estimate. Rather, we have based it partly on prior research indicating that inhalation is a primary route of exposure to semivolatile insecticides after residential use (Pang et al. 2002; Whitmore et al. 1994). Further, although dietary intakes can also be an important exposure route (Clayton et al. 2003; Morgan et al. 2005), we think it unlikely that dietary exposures changed appreciably over the course of the study. The U.S. EPA regulations were aimed principally at eliminating residential use of chlorpyrifos, and it is still widely used in agriculture including on food crops. In addition, we have based our assumption on the high correlations that have been seen in prior research between indoor and personal air levels of chlorpyrifos and other semivolatile insecticides and levels in carpet dust, hand wipes, and surfaces in the home (Gordon et al. 1999; Lewis 2004; Pang et al. 2002). A recent aggregate assessment study of chlorpyrifos exposures among children also found a high correlation between exposures via the inhalation and dermal routes (Morgan et al. 2005). Thus, it is likely that the decrease in chlorpyrifos in personal and indoor air samples we found here also reflects a decrease in residential exposures via dermal absorption and nonintentional ingestion, but we did not measure the exact magnitude of the change in exposures via these other routes.

In conclusion, our prior research has shown that the U.S. EPA restrictions on residential uses of chlorpyrifos were remarkably effective at eliminating sales of the insecticide in inner-city communities in New York City (Carlton et al. 2004) and at reducing personal and indoor air levels among subjects in the present cohort (Whyatt et al. 2007). The present study provides a vivid illustration of the efficacy of the restrictions to reduce the internal dose of chlorpyrifos to mother and fetus during pregnancy.

## Figures and Tables

**Table 1:** Cohort demographics and the number of women (%) reporting sightings of pests and use of pest control in the home over the final 2 months of pregnancy (*n* = 102).

Characteristic^a^	No. (%)
Age [years (mean ± SE)]	25.2 ± 4.9
Ethnicity	
African American	31 (31)
Dominican	68 (68)
Other	1 (1)
Marital status	
Never married	69 (69)
Married^b^	24 (24)
Separated, widowed, divorced	7 (7)
Education	
< High school degree	46 (46)
High school diploma or GED	33 (33)
Some college (< 4 years)	20 (20)
College degree (4 years)	1 (1)
Income	
< $10,000	36 (42.9)
$10,000–$30,000	36 (42.9)
> $30,000	12 (14.3)
Total number with pest sightings	90 (91)
Sightings of cockroaches	79 (80)
Sighting of rodents (mice and rats)	51 (51.5)
Sightings of other pests	21 (21)
Total number using pest control	59 (60)
Used sticky traps, gels, and baits only	31 (32)
Used can sprays, pest bombs, or sprays by exterminator (with or without other methods)	28 (29)

GED, general educational diploma.

a Missing values: maternal age (*n* = 3), ethnicity (*n* = 2), marital status (*n* = 2), education (*n* = 2), income (*n* = 18), pest sightings (*n* = 3), and pest control (*n* = 4).

b Includes women living as married with same partner > 7 years.

**Table 2:** TCPy levels in repeat maternal spot urine samples between the 34th week of pregnancy and delivery and in a maternal urine sample after delivery, before adjusting for creatinine and after adjusting for creatinine.

		Percentile					
Sample	No. > LOD/total (%)^a^	10th	25th	50th	75th	90th	95th
Before adjusting for creatinine (μg/L)							
1st prenatal	49/95 (52)	< LOD	< LOD	0.46	2.1	3.7	7.8
2nd prenatal	40/83 (48)	< LOD	< LOD	< LOD	1.3	2.6	3.5
3rd prenatal	30/54 (56)	< LOD	< LOD	0.57	2.4	5.2	6.9
4th prenatal	13/21 (62)	< LOD	< LOD	0.61	3.3	5.3	6.8
Postnatal	29/73 (40)	< LOD	< LOD	< LOD	1.02	3.3	4.8
After adjustment for creatinine (μg/g creatinine)^b^							
1st prenatal	48/91 (53)	< LOD	< LOD	0.71	2.0	3.5	6.2
2nd prenatal	40/83 (48)	< LOD	< LOD	< LOD	1.7	3.1	4.2
3rd prenatal	30/53 (57)	< LOD	< LOD	0.83	2.1	5.2	6.4
4th prenatal	13/19 (68)	< LOD	< LOD	1.3	4.3	5.8	6.8
Postnatal	29/69 (42)	< LOD	< LOD	< LOD	1.5	2.9	4.5

a LOD = 0.26 μg/L.

b Creatinine was missing for 11 urine samples.

**Table 3:** Spearman’s rank correlation of TCPy levels in repeat spot maternal urine samples during pregnancy and after delivery (μg/g creatinine) in all subjects and in subjects enrolled in 2001–2002.

Sample	1st prenatal	2nd prenatal	3rd prenatal	4th prenatal
All subjects				
Postpartum	0.25 (*n* = 61)	0.36** (*n* = 56)	0.25 (*n* = 39)	0.31 (*n* = 15)
4th prenatal	0.49* (*n* = 18)	0.51* (*n* = 19)	0.29 (*n* = 19)	
3rd prenatal	0.25 (*n* = 51)	0.69** (*n* = 52)		
2nd prenatal	0.43** (*n* = 79)			
Subjects enrolled in 2001–2002				
Postpartum	0.23 (*n* = 41)	0.18 (*n* = 40)	−0.18 (*n* = 29)	0.31 (*n* = 15)
4th prenatal	0.49* (*n* = 18)	0.44 (*n* = 18)	0.23 (*n* = 18)	
3rd prenatal	0.44** (*n* = 36)	0.43** (*n* = 36)		
2nd prenatal	0.56** (*n* = 79)			

* *p* < 0.05,

** *p* < 0.001.

**Table 4:** Spearman’s rank correlation between chlorpyrifos in 2-week integrated indoor air samples collected between the 32nd–34th week of pregnancy through delivery and TCPy levels in spot maternal urine samples collected every 2 weeks over the same time period, among all subjects and among subjects enrolled in 2001–2002.

	Metabolite levels in maternal urine samples (μg/g creatinine)				
Indoor air (ng/m^3^ )	1st prenatal	2nd prenatal	3rd prenatal	4th prenatal	Two-month average
All subjects (week of pregnancy)					
32nd–34th	*r* = 0.38** (*n* = 85)				
34th–36th		*r* = 0.28* (*n* = 76)			
36th–38th			*r* = 0.33* (*n* = 47)		
38th–40th				*r* = 0.45* (*n* = 19)	
2-month average					*r* = 0.31* (*n* = 95)
Subjects enrolled 2001–2002 (week of pregnancy)					
32nd–34th	*r* = 0.44** (*n* = 56)				
34th–36th		*r* = 0.33* (*n* = 51)			
36th–38th			*r* = 0.30 (*n* = 35)		
38th–40th				*r* = 0.51* (*n* = 18)	
2-month average					*r* = 0.44** (*n* = 57)

* *p* = 0.05;

** *p* = 0.001.

**Table 5:** Chlorpyrifos levels in maternal and umbilical cord blood and TCPy levels in postpartum meconium collected after delivery.

		Percentile					
Sample	No. > LOD/total (%)^a^	10th	25th	50th	75th	90th	95th
Maternal blood (pg/g)^a^	13/92 (14)	< LOD	< LOD	< LOD	< LOD	1.5	2.5
Umbilical cord blood (pg/g)^a^	8/65 (12)	< LOD	< LOD	< LOD	< LOD	2.3	2.5
Postpartum meconium (ng/g)^b^	23/83 (28)	< LOD	< LOD	< LOD	0.44	0.62	0.77

a LOD = 0.5–1 pg/g.

b LOD = 0.2 ng.

**Table 6:** Spearman’s rank correlation of TCPy levels in meconium and chlorpyrifos in maternal and umbilical cord blood and TCPy in spot urine samples collected from the mother during pregnancy and after delivery.

Sample	TCPy levels in meconium (ng/g)
Chlorpyrifos in blood samples (pg/g)	
Maternal blood	*r* = 0.25, *p* = 0.03, *n* = 79
Umbilical cord blood	*r* = 0.33, *p* = 0.01, *n* = 56
TCPy in maternal urine (μg/g creatinine)	
1st prenatal	*r* = 0.22, *p* = 0.06, *n* = 74
2nd prenatal	*r* = 0.35, *p* = 0.003, *n* = 71
3rd prenatal	*r* = 0.28, *p* = 0.06, *n* = 45
4th prenatal	*r* = 0.40, *p* = 0.10, *n* = 17
Average prenatal	*r* = 0.31, *p* = 0.006, *n* = 78
After delivery	*r* = 0.32, *p* = 0.01, *n* = 60
